# JWA suppresses proliferation in trastuzumab-resistant breast cancer by downregulating CDK12

**DOI:** 10.1038/s41420-021-00693-9

**Published:** 2021-10-22

**Authors:** Yan Liang, Chao Qian, Yinghong Xie, Xiang Huang, Junjie Chen, Yanlin Ren, Ziyi Fu, Yongfei Li, Tianyu Zeng, Fan Yang, Jianwei Zhou, Wei Li, Yongmei Yin, Changqing Wang

**Affiliations:** 1 Department of Oncology, The First Affiliated Hospital of Nanjing Medical University, Nanjing, China; 2 Department of General Surgery, Sir Run Run Hospital, Nanjing, China; 3 Clinical Medical Research Center, Affiliated Hospital of Nantong University, Nantong, China; 4 Nantong Center for Disease Control and Prevention, Nantong, China; 5 Laboratory of Breast Disease Department, The First Affiliated Hospital of Nanjing Medical University, Nanjing, China; 6 Department of General Surgery, Jiangsu Provincial Hospital of Traditional Chinese Medicine, Nanjing, China; 7 Department of Molecular Cell Biology & Toxicology, Center for Global Health, School of Public Health, Nanjing Medical University, Nanjing, China; 8 School of Health Policy and Management, Nanjing Medical University, Nanjing, China

**Keywords:** Genetics, Breast cancer

## Abstract

Breast cancer is the most common cancer worldwide. JWA is a microtubule-associated protein that has been identified as a tumor suppressor, and its downregulation in tumors is an independent adverse prognostic factor. The objective of this study was to explore the expression, regulation, and mechanism of JWA in trastuzumab-resistant breast cancers. In this study, we found that JWA expression was lower in trastuzumab-resistant breast cancers than that in trastuzumab-sensitive breast cancers. Furthermore, it was confirmed that overexpression of JWA inhibited proliferation and promoted apoptosis in trastuzumab-resistant breast cancers both in vitro and in vivo. In addition, the low expression of JWA in trastuzumab-resistant breast cancers is associated with a poor prognosis. Combining RNA-sequence datasets and next-generation sequencing, it was found that JWA negatively regulated CDK12, and was involved in the G1-to-S transition of the cell cycle. It has been reported that CDK12 drives breast cancer initiation and induces trastuzumab resistance. Taken together, high expression of JWA could inhibit the growth of trastuzumab-resistant breast cancer, and JWA is a potential predictive marker for trastuzumab resistance. In addition, targeted therapy with JWA may be a novel therapeutic strategy to improve the survival rate of trastuzumab-resistant breast cancer.

## Introduction

Breast cancer is the leading cause of cancer-related death among women worldwide [1]. Human epidermal growth factor receptor 2 (HER2/ErbB2) is a member of the epidermal growth factor receptor family, and HER2-positive (HER2+) breast cancer accounts for approximately 20% of the histological types of breast cancer with a poor prognosis [2]. Trastuzumab was the first HER2-targeted monoclonal antibody therapy, and it has since become the most commonly used treatment for HER2+ breast cancer [3]. However, mounting clinical evidence has revealed that not all patients with HER2 overexpression benefit from trastuzumab therapy, and more than 50% of patients develop resistance to trastuzumab during treatment [4, 5]. Trastuzumab resistance is defined as progression at first radiological reassessment at 8–12 weeks or within 3 months after first-line trastuzumab with or without chemotherapy in the metastatic setting, or as a new recurrence diagnosed within 12 months of adjuvant trastuzumab therapy [6]. Therefore, the treatment of HER2+ breast cancer is often hindered due to resistance to HER2-targeted therapies. There are several mechanisms associated with trastuzumab resistance, including the inhibition of downstream signaling pathways and bypassing of HER2 via ErbB-receptor crosstalk [7, 8].

JWA, also known as adenosine diphosphate (ADP) ribosylation-like factor 6 interacting protein 5 [9], is a multifunctional microtubule-associated protein involved in DNA damage repair, apoptosis, and cell differentiation in a variety of physiological environments [9–12]. JWA has been found to inhibit invasion, adhesion, and angiogenesis in triple-negative breast cancer, melanoma, gastric cancer, and liver cancer [13–18]. High JWA expression has also been shown to be a good prognostic indicator, and JWA enhances apoptosis of certain chemotherapeutic agents [19, 20]. The clinical significance of JWA in trastuzumab-resistant breast cancer has not been fully elucidated; however, there is increasing evidence supporting its role in tumor inhibition.

Cyclin-dependent kinase 12 (CDK12), a member of the CDK family, is located on chromosome 17 [21]. CDK12 plays a key role in many cellular processes and is mutated or overexpressed in various types of cancer. Indeed, its overexpression in tumors suggests that CDK12 may have carcinogenic properties [22]. High CDK12 activity has been shown to accelerate tumor progression and treatment resistance [22]. In approximately 71% of HER2+ breast cancers, the HER2 amplicon also contains the CDK12 gene [23, 24]. This plays a role in tumor promotion in HER2+ breast cancer with high CDK12 expression associated with poor overall survival (OS) and disease-free survival [25–27]; CDK12 has also been shown to play an anticancer role in triple-negative breast cancer [21]. CDK12 is associated with the expression of DNA damage response (DDR) genes [28] and has been considered as a drug target for HER2+ breast cancer [23].

## Materials and methods

### Patient samples

A total of 32 patients with HER2+ breast cancer and who were treated with trastuzumab were enrolled in this study from the First Affiliated Hospital of Nanjing Medical University from 2011 to 2019. Half of them were sensitive to trastuzumab and the rest of them were resistant. Biopsy samples were obtained from them. In addition, cancerous tissue samples of the 16 trastuzumab-resistant breast cancer patients mentioned above were collected for next-generation sequencing (NGS). General clinical information and detailed pathological records were obtained for all patients. Institutional review board approval was obtained, as was written informed consent from the patients.

### Cell culture

Three human HER2+ breast cancer cell lines (JIMT1, SKBR3, and BT474) were obtained from the American Type Culture Collection (ATCC) (Manassas, VA, USA). All cell lines were cultured in Dulbecco’s modified Eagle’s medium (Gibco, Thermo Fisher Scientific, Waltham, MA, USA) supplemented with 10% fetal bovine serum (FBS), 100 U/mL penicillin sodium, and 100 µg/mL streptomycin sulfate in humidified air at 37 °C with 5% CO2 according to ATCC protocols. Trastuzumab (Roche, Switzerland) was dissolved in phosphate-buffered saline (PBS) prior to use. BT474 cells resistant to trastuzumab treatment (named as BT474-Tr) were created by treating parental BT474 cells with increasing concentrations of trastuzumab for approximately 6 months. These BT474-Tr cells were subsequently maintained in the presence of trastuzumab (15 µg/mL). These cell lines were routinely tested for mycoplasma.

### Plasmids, siRNAs, and cell transfection

Lentivirus‐overexpressing JWA particles and overexpressing control particles, overexpressing CDK12 and control plasmids, JWA siRNA, and negative control siRNA were purchased from Shanghai Genechem (Shanghai, China). CDK12 siRNA and negative control siRNA were obtained from Generay (Shanghai, China). The DNA plasmids or siRNA were transfected into cells using Lipofectamine 3000 (Invitrogen, Carlsbad, CA, USA) according to the manufacturer’s instructions.

### RNA extraction and real-time polymerase chain reaction (RT-PCR) assay

Total RNA samples were extracted from cell lines using Trizol reagent (Gibco BRL, Gaithersburg, MD, USA) according to the manufacturer’s protocol. The complementary DNA (cDNA) templates were reverse transcribed using a reverse transcription kit and SYBR Green Master Mix kit (Takara, Otsu, Japan). The cDNA was amplified using the following primers: 5-GGAGGAGTCATTGTGGTGC-3 (forward) and 5-GAAGTCTCAGGGATGCGTG-3 (reverse) for JWA; 5-GGGACAAGTACTGAGCCTGT-3 (forward) and 5-CTCTGGGTTGGAGTGGATGT-3 (reverse) for CDK12; and 5-GCCGGTGCTGAGTATGTC-3 (forward) and 5-CTTCTGGGTGGCAGTGAT-3 (reverse) for GAPDH. Quantitative RT-PCR was carried out using an ABI Prism 7900 Sequence Detection System (Applied Biosystems, Foster City, CA, USA), and relative levels were normalized to that of GAPDH.

### Western blot

All protein samples were isolated from cell lines and tissues, cell samples were lysed in lysis buffer (50 mM Tris, pH 7.4; 150 mM NaCl; 1% NP-40; 0.5% sodium deoxycholate; 0.1% sodium dodecyl sulfide (SDS); and the protease inhibitor, 1 mM phenylmethylsulfonyl fluoride (PMSF)), and tissue samples were used for tissue protein extraction reagent (Thermo Fisher Scientific). Protein concentration was measured using a bicinchoninic acid (BCA) protein assay kit (Thermo Fisher Scientific). Proteins (40 μg) were processed for analysis. The antibodies used for the analysis were as follows: anti-JWA (1:100; produced in our laboratory), anti-CDK12 (1:1000; Proteintech, Rosemont, IL, USA), anti-Bcl-2 (1:2000; Abcam, Cambridge, UK), anti-Caspase 3 (1:500; Abcam), anti-PCNA (1:1000; Abcam), anti-BAX (Ser448, 1:1000; Proteintech). The membrane was blocked with 5% milk powder for 1 h before incubation with primary antibodies and species-specific secondary antibodies. After washing with PBS plus Tween^®^ detergent, bands were visualized with an enhanced chemiluminescence detection kit and exposed to an X-ray film.

### Cell viability assay

Each cell type was prepared as described above, and a total of 2000 cells with corresponding treatment were seeded in 96-well plates with three replicates with an initial homogenous confluence. At specific time points, a cell counting Kit-8 (CCK-8; APExBIO Technology, Houston, TX, USA) was used for cell proliferation assays. Growth curves were constructed using the normalized confluence values of each well.

### Colony formation assay

Cells were plated in six-well plates, cultured in a medium containing FBS, and incubated for approximately 15–18 days. The plates were gently washed with PBS and fixed with methanol for 30 min. Plates were then washed again with PBS, and colonies were stained with crystal violet for 2 h at room temperature (25 °C). After staining, plates were washed with water to rinse off the excess stain. Following image acquisition, the percent colony area was automatically calculated using an ImageJ plugin.

### Immunohistochemistry (IHC)

Tumor tissues were harvested and placed in 10% formalin buffer. Sections were deparaffinized, followed by antigen retrieval with sodium citrate buffer and blocking of endogenous peroxidase activity with 3% H2O2. Slices were then incubated with primary antibody anti-JWA (1:100), anti‐CDK12 (1:200), anti-Ki67 (1:500; Abcam), and anti‐Caspase-3 (ab32351, 1:100) in blocking buffer overnight at 4 °C. Slides were washed three times for 5 min each using PBS and incubated with secondary antibodies diluted 1:1000 at room temperature for 1 h. The presence of a brown chromogen in the cytoplasm indicated positive immunoreactivity. The immunostaining intensity of each sample was graded as negative = 0, weak = 1, moderate = 2, or strong = 3. The percentage of positively stained cells was assessed as follows: 0 (no tumor cells stained), 1(1%–25%), 2 (26%–50%), 3 (51%–75%), or 4 (76%–100%), and the score was calculated as the intensity score multiplied by the percentage of stained cells. Scores > 4 were defined as JWA-high, and scores ≤ 4 were considered JWA-low.

### Flow cytometry

Transfected cells were seeded in a six-well plate. After 2 days, the cells were washed with PBS and fixed in 70% ethanol overnight at 4 °C. The cells were then stained with propidium iodide (BD Biosciences, San Jose, CA, USA) for 30 min and analyzed using a flow cytometer.

### Hoechst 33342 staining to detect cell apoptosis

Hoechst 33342 (Beyotime Biotechnology, Haimen, China) was used to identify apoptotic cells. JIMT1 and BT474-Tr cells overexpressing JWA and control particles were cultured for 3 days. The cells were washed with PBS and then stained with Hoechst 33342 DNA-binding dye (10 mg/L) in the dark at 37 °C for 15 min. Finally, the cells were washed with PBS and observed under a fluorescence microscope (Leica Camera AG, Wetzlar, Germany).

### In vivo nude mouse model

Our study was approved by the Ethics Committee of Nanjing Medical University (IACUC-1811067). JIMT1 cells were inoculated subcutaneously into the flanks of 6-week-old female BALB/c nude mice obtained from the Model Animal Research Center of Nanjing Medical University (Nanjing, China). Mice were randomly divided into two groups (*n* = 8/group), and 5 × 10^6^ JIMT1 cells infected with Flag-JWA or Flag-con were injected into nude mice, and mice experiments were not blind. The diameters of the tumors were measured three times a week using digital calipers, and tumor volume was calculated as [length × (width)^2^]/2. Mice were resected after 32 days, and the weights of the neoplasms were measured. Total RNA samples were extracted from mouse tumor tissue specimens using Trizol reagent (Gibco BRL, Gaithersburg, MD, USA) according to the manufacturer’s protocol. Tumor tissues were then fixed for pathological slides with 4% paraformaldehyde fixation followed by IHC, and the other tissues were flash-frozen at −80 °C for immunoblotting.

### Statistical analysis

Results are presented as the mean ± standard deviation. GraphPad Prism 8 was used for all calculations and plotting of graphs, and *P* < 0.05 was considered as the threshold for significance. The applied statistical analysis is shown in the figure legends.

## Results

### JWA is downregulated in trastuzumab-resistant breast cancer and is associated with patient prognosis

**Fig. 1:**
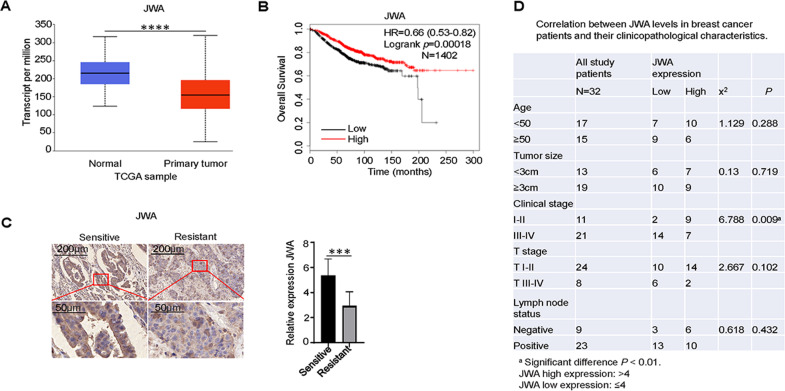
JWA is downregulated in trastuzumab-resistant breast cancer and is associated with patient prognosis. **A** The levels of JWA in normal and breast cancer samples in TCGA database. **B** Correlation between JWA levels and overall survival. **C** IHC analysis of JWA expression in trastuzumab sensitive and resistant breast cancer samples, and JWA expression was lower in trastuzumab-resistant tissues. The cytoplasmic staining results were evaluated based on the percentage of positive cells and the intensity of staining. **D** Clinical association studies found that JWA expression was significantly associated with clinical stages (*P* < 0.05). Statistical analysis was performed using chi-square. ***P < 0.001, ****P < 0.0001.

The analysis showed that the expression of JWA in breast cancer samples (*n* = 1097) was significantly lower than that in normal samples (*n* = 114) (http://ualcan.path.uab.edu/analysis.html) (Fig. 1A). In all breast cancers, patients with high JWA expression had significantly longer OS than those with low JWA expression. Lower expression of JWA was significantly associated with poor patient prognosis (http://kmplot.com/analysis/index.php?p=background) (Fig. 1B). JWA inhibits cancer cell invasion and tumorigenesis in various human cancers. To assess the underlying role of JWA in trastuzumab-resistant breast cancer, we compared its expression in 16 paired trastuzumab-resistant and -sensitive breast cancer tissues. JWA expression was significantly decreased in trastuzumab-resistant breast cancer samples compared to that in trastuzumab-sensitive breast tissues (Fig. 1C). A total of 32 breast cancer tissues were evaluated to determine the pathologic correlation between JWA expression and breast cancer progression using established breast cancer prognostic factors (Fig. 1D). This indicated that the expression of JWA was higher in patients with clinical stages I-II than in those with stages III-IV. Therefore, JWA was highly expressed in patients with earlier clinical stages (*P* = 0.009). However, JWA was not significantly associated with age, tumor size, T stage, and lymph node status in patients with HER2+ breast cancer. Taken together, these findings suggest that JWA could serve as an effective predictor of malignant progression in HER2+ breast cancer.

### JWA suppresses proliferation in trastuzumab-resistant breast cancer cells In vitro

**Fig. 2:**
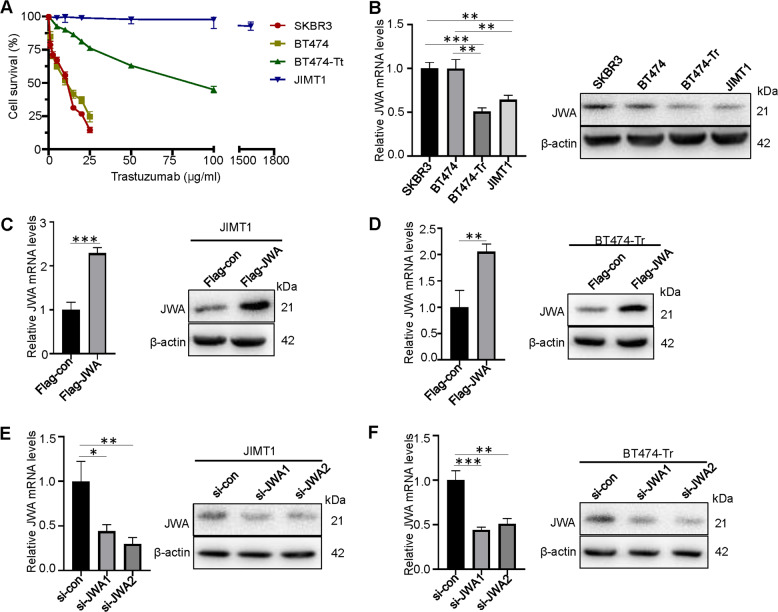
JWA is low expressed in trastuzumab-resistant breast cancer cells. **A** Sensitivity to trastuzumab was measured in different HER2+ breast cancer cells by CCK-8. **B** JWA was measured in different HER2+ breast cancer cells by qRT-PCR and Western blot. **C**, **D** JWA was measured by qRT-PCR and Western blot in JIMT1 and BT474-Tr cells stably expressing JWA. **E**, **F** JWA was measured by qRT-PCR and Western blot in JIMT1 and BT474-Tr cells transfected with two independent JWA siRNAs. *P < 0.05, **P < 0.01, ***P < 0.001.

**Fig. 3:**
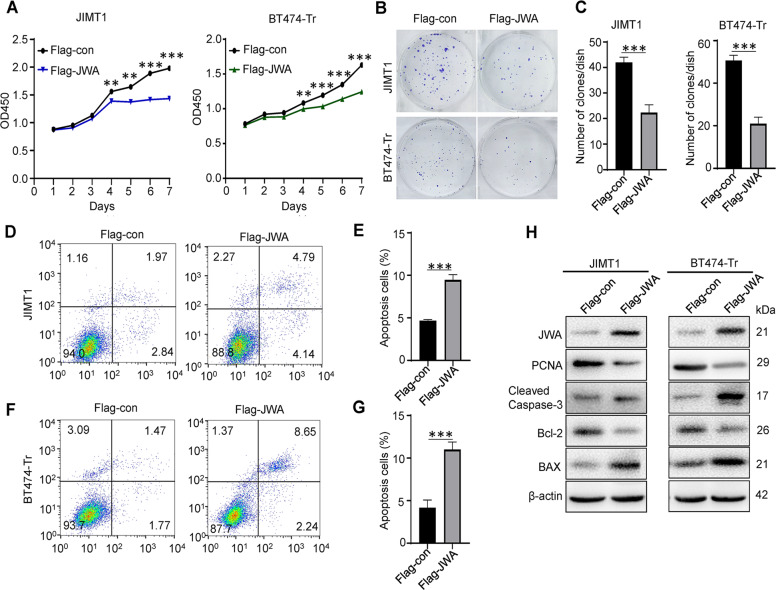
JWA suppresses proliferation and promotes apoptosis in trastuzumab-resistant breast cancer cells in vitro. **A** Overexpression of JWA significantly increased the inhibition rate of tumor cells in JIMT1 and BT474-Tr cells by CCK-8. **B**, **C** Overexpression of JWA inhibited cell proliferation in JIMT1 and BT474-Tr cells as determined by colony formation assays. **D**, **E** Apoptotic cells increased after overexpression of JWA in JIMT1 cells. **F**, **G** Apoptotic cells increased after overexpression of JWA in BT474-Tr. **H** Overexpression of JWA in JIMT1 and BT474-Tr cells inhibited cell proliferation and promoted apoptosis as assessed by Western blot. Statistical analysis was performed using Student’s t-test. *P < 0.05, ** *P* < 0.01, *** *P* < 0.001, ns statistical difference.

We tested the sensitivity of four HER2+ breast cancer cell lines (SKBR3, BT474, BT474-Tr, and JIMT1) to trastuzumab by CCK-8, and found that the IC50 values of SKBR3 and BT474 were between 10 and 15 μg/mL, and the IC50 of BT474-Tr was much higher than that of the former. Therefore, it was confirmed that BT474-Tr was an induced trastuzumab-resistant cell line, while JIMT1 was a natural trastuzumab-resistant cell line (Fig. 2A). To determine the role of JWA in HER2+ trastuzumab-resistant breast cancer, we identified the expression of JWA in four HER2+ breast cancer cell lines by qRT-PCR and western blotting. JWA mRNA and protein were found to be downregulated in trastuzumab-resistant cells compared with trastuzumab-sensitive cells (Fig. 2B). Thus, to investigate the functional roles of JWA in trastuzumab-resistant breast cancer cells, we established stable cell lines (JWA) via lentiviral infection in JIMT1 and BT474-Tr cells and knocked down endogenous JWA via two independent siRNAs in the JIMT1 and BT474-Tr cells (Fig. 2C−F). The CCK-8 results showed that the growth rate of the Flag-JWA group was markedly slower than that of the Flag-con group in the JIMT1 and BT474-Tr cells (Fig. 3A). The colony numbers were reduced after JWA overexpression in the JIMT1 and BT474-Tr cells by colony formation assays (Fig. 3B, C). Stable overexpression of JWA in the JIMT1 and BT474-Tr cells significantly inhibited the proliferation and colony formation abilities of trastuzumab-resistant breast cancer cells compared with those of the control cells. Knockdown of JWA substantially promoted the proliferation and colony formation abilities of trastuzumab-resistant breast cancer cells compared to those of the control cells (Supplementary Fig. 1A, B). Moreover, the results of western blotting showed that stable overexpression of JWA significantly suppressed the expression of PCNA, indicating that overexpression of JWA inhibited proliferation (Fig. 3H). These data indicate that JWA upregulation inhibits the proliferation of trastuzumab-resistant breast cancer cells.

### JWA promotes apoptosis in trastuzumab-resistant breast cancer cells In vitro

Stable overexpression of JWA increased the apoptotic abilities of JIMT1 and BT474-Tr cells (Fig. 3D−G) (Supplementary Fig. 1C, D). Western blot analysis showed that BAX and Cleaved Caspase-3 expression increased and Bcl-2 expression decreased following overexpression of JWA (Fig. 3H). The above results revealed that the stable overexpression of JWA significantly promoted apoptosis.

### JWA negatively regulates CDK12

**Fig. 4:**
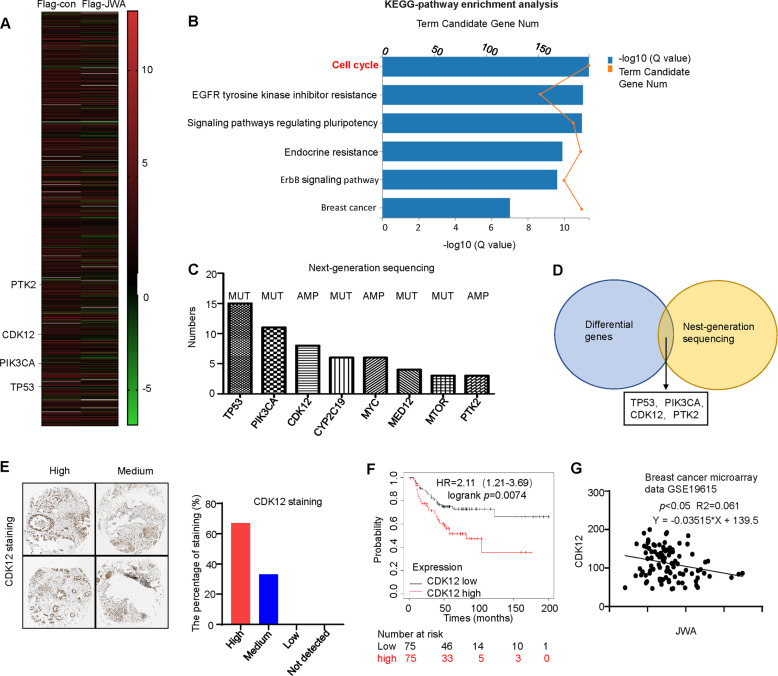
JWA negatively regulates CDK12. **A** Some genes altered significantly after overexpression of JWA by RNA-seq. **B** The results of KEGG-pathway enrichment analysis showing the cell cycle pathway had the greatest significance. **C** Somatic genetic alterations identified in 16 trastuzumab-resistant breast cancer patients subjected to NGS. **D** The intersection frequencies of genetic changes and NGS were ranked top 4 in the order of TP53, PIK3CA, CDK12, PTK2. **E** The IHC of 12 breast cancer patients through The Human Protein Atlas database, and CDK12 was highly expressed in eight patients and that was moderately expressed in four patients; (×40). **F** Survival curves showing a comparison of survival rates in patients with HER2+ breast cancer with low and high CDK12 expression. **G** Scatter plot showing the correlation between JWA and CDK12 expression in the breast cancer microarray data GSE19615. MUT mutation; AMP amplification.

By combining RNA-sequence (RNA-seq) datasets to identify differential gene expression with high confidence, we found that TP53, PIK3CA, CDK12, and PTK2 expression were significantly increased. However, it decreased after JWA overexpression in JIMT1 cells (Fig. 4A). Through KEGG pathway enrichment analysis, we found that the cell cycle pathway was the most significant (Fig. 4B). It has been reported that CDK12 regulates multiple biological processes, including cell cycle progression [29]. A total of 16 patients with trastuzumab-resistant breast cancer were tested using NGS in our hospital, in these patients, the frequency of genetic changes was ranked as the top four in the order of TP53, PIK3CA, CDK12 and CYP2C19 (Fig. 4C). The intersection frequencies of genetic changes and NGS were TP53, PIK3CA, CDK12 and PTK2 (Fig. 4D). Therefore, we believe that JWA is likely to inhibit breast cancer growth by downregulating CDK12. Choi [25] reported that CDK12 is a novel regulatory factor of tumor stem cells, which can promote tumor initiation and induce anti-HER2 resistance in breast cancer. We analyzed CDK12 expression in 12 breast cancer patients using the Human Protein Atlas database (https://www.proteinatlas.org/). Among the 12 breast cancer patients, CDK12 was highly expressed in eight patients and moderately expressed in four patients (Fig. 4E). Survival analysis of the HER2+ breast cancer patients showed that the survival time of patients with high expression of CDK12 was significantly lower than that of patients with low expression, with the difference being statistically significant (*P* = 0.0074) (Fig. 4F). Breast cancer microarray data (GSE19615) revealed that high expression of JWA was correlated with low levels of CDK12 in breast cancer (Fig. 4G).

### CDK12 is involved in the promotion of proliferation and inhibition of apoptosis in trastuzumab-resistant breast cancer

**Fig. 5:**
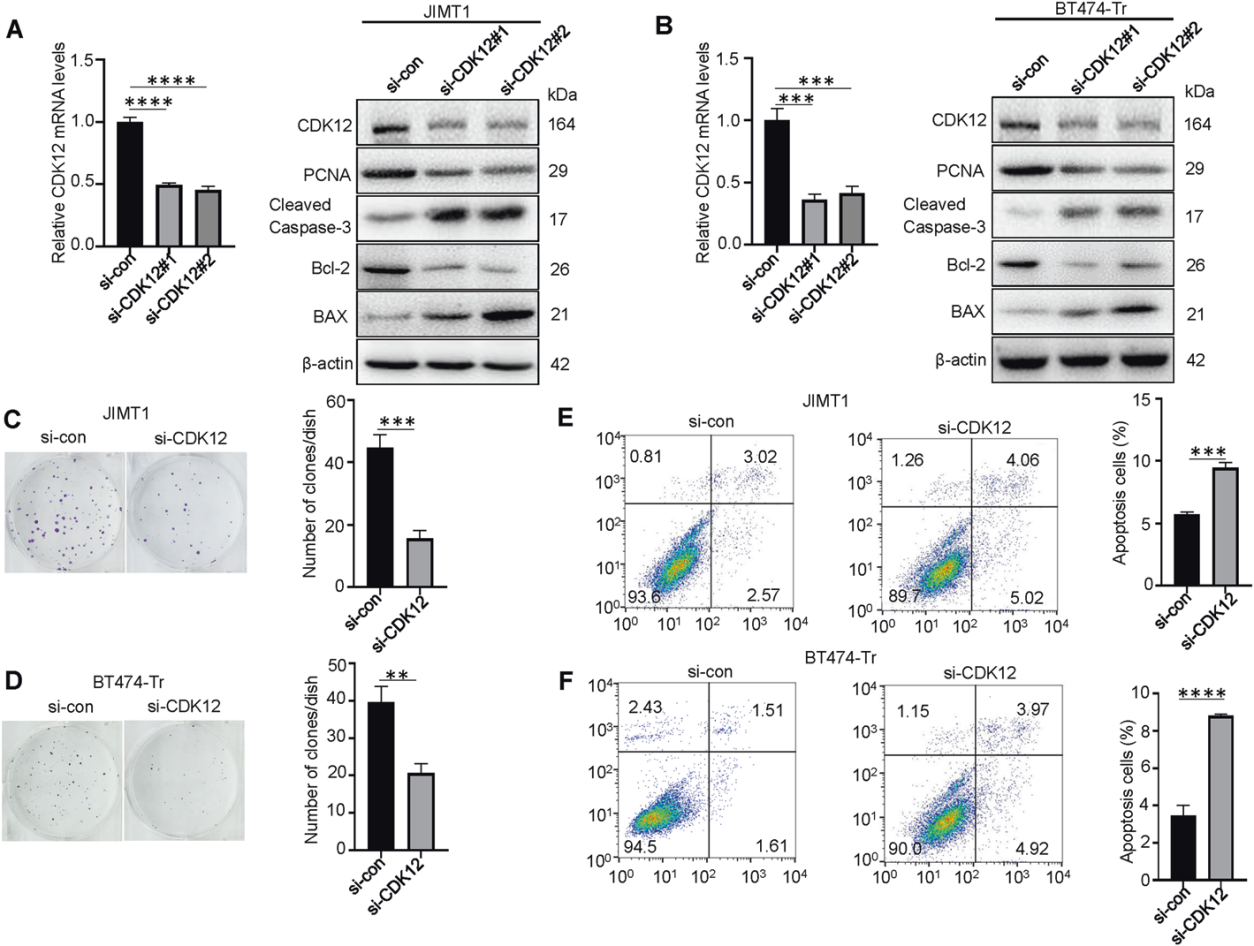
CDK12 is involved in the promotion of proliferation and inhibition of apoptosis in trastuzumab-resistant breast cancer. **A**, **B** qRT–PCR results showing levels of CDK12 expression in JIMT1 and BT474-Tr cells transfected with CDK12 siRNAs, respectively. Protein levels of PCNA, Cleaved Caspase-3, Bcl-2, BAX in JIMT1 and BT474-Tr cells transfected with CDK12 siRNAs. β-actin served as the internal control. **C**, **D** Interference with CDK12 siRNAs in JIMT1 and BT474-Tr cells impacted cell proliferation as determined by colony formation assays. **E**, **F** Apoptotic cells increased after knocking down of CDK12 in JIMT1 and BT474-Tr cells. **P < 0.01, ***P < 0.001, ****P < 0.0001.

To investigate the functional roles of CDK12 in trastuzumab-resistant breast cancer progression, we knocked down CDK12 via two independent siRNAs in JIMT1 and BT474-Tr cells (Fig. 5A, B). Knockdown of CDK12 decreased the levels of PCNA and Bcl-2, and increased the levels of Cleaved Caspase-3 and BAX (Fig. 5A, B). Similar effects were observed in the colony formation assay, which showed that the number of clones decreased after knockdown of CDK12, so knockdown of CDK12 significantly inhibited the proliferation of JIMT1 and BT474-Tr cells (Fig. 5C, D). Knockdown of CDK12 clearly increased the apoptosis abilities of JIMT1 and BT474-Tr cells (Fig. 5E, F).

### JWA inhibits proliferation and facilitate apoptosis in trastuzumab-resistant breast cancer via downregulating CDK12 In vitro

**Fig. 6:**
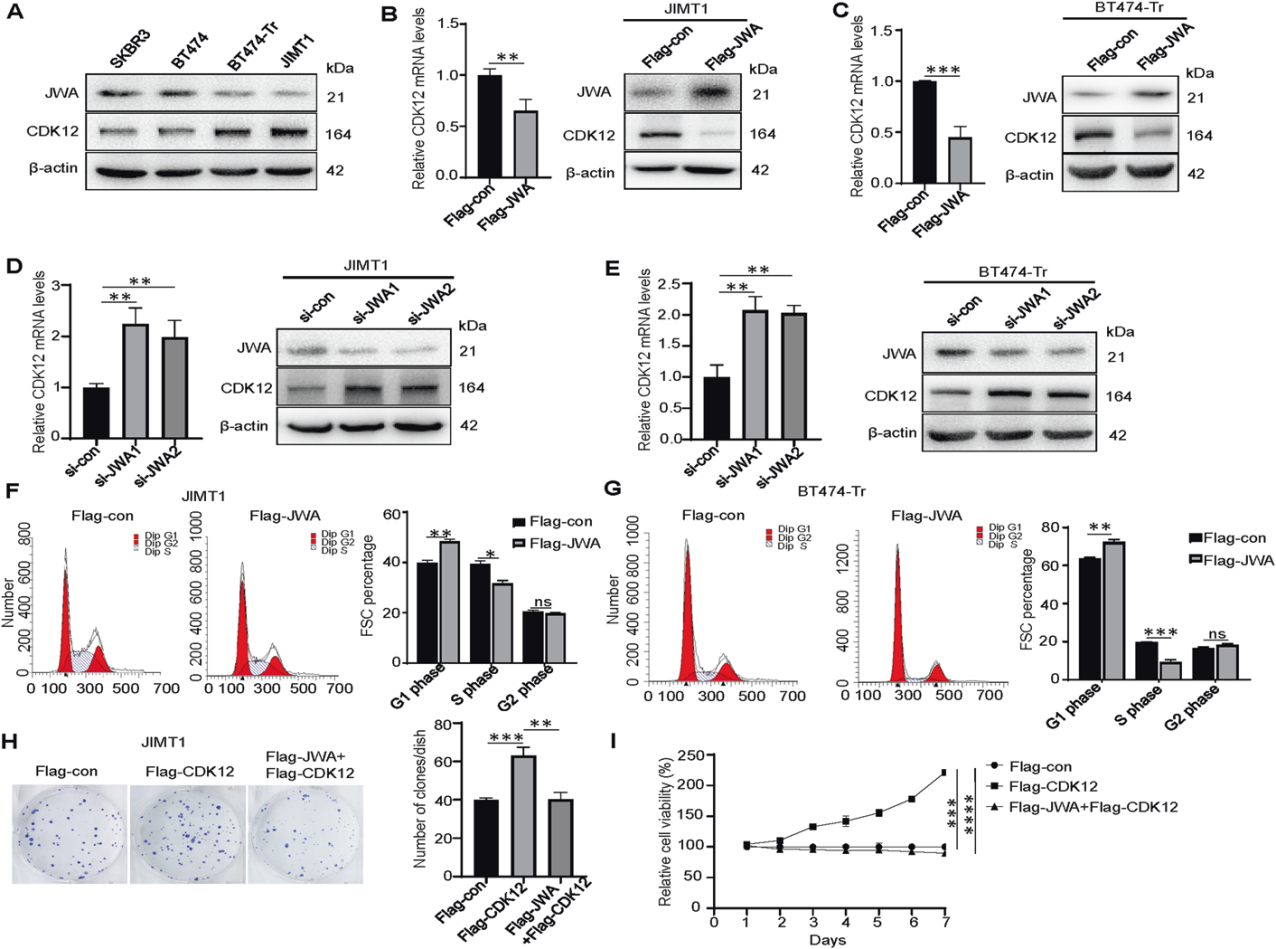
JWA inhibits proliferation and facilitates apoptosis in trastuzumab-resistant breast cancer via downregulating CDK12 in vitro. **A** JWA and CDK12 were measured in different HER2+ breast cancer cells by Western blot. **B**, **C** Overexpression of JWA significantly downregulated CDK12 expression in JIMT1 and BT474-Tr cells by qRT-PCR and Western blot. **D**, **E** Knocking down of JWA significantly upregulated CDK12 expression in JIMT1 and BT474-Tr cells by qRT-PCR and Western blot. **F**, **G** Overexpression of JWA in JIMT1 and BT474-Tr cells was detected by flow cytometer, and it arrested cell cycle at the G1/S phase in trastuzumab-resistant breast cancer cells. **H**, **I** Overexpression of CDK12 in JIMT1 cell promoted proliferation, and further overexpression of JWA inhibited proliferation as determined by colony formation assays and CCK8. *P < 0.05, **P < 0.01, ***P < 0.001, ****P < 0.0001.

CDK12 protein were found to be downregulated in trastuzumab-sensitive cells compared with trastuzumab-resistant cells (Fig. 6A). To gain insight into the underlying mechanism of JWA in trastuzumab-resistant breast cancer, we found that overexpression of JWA significantly downregulated CDK12 levels in JIMT1 and BT474-Tr cells (Fig. 6B, C). Conversely, knockdown of JWA significantly upregulated CDK12 levels (Fig. 6D, E). Flow cytometry analysis showed that JWA overexpression increased the percentage of cells in the G1 phase and decreased the percentage of cells in the S phase (Fig. 6F, G), indicating that JWA might inhibit the G1-to-S phase transition in trastuzumab-resistant breast cancer cells.

We performed a reactivation test with JIMT1 using colony formation assay and CCK-8. The results showed that the growth rate of the Flag-CDK12 group was markedly higher than that of the Flag-con group in JIMT1 cells, and the growth rate of the JWA and CDK12 overexpression group was significantly slower than that of the Flag-CDK12 group. Therefore, overexpression of CDK12 promoted proliferation, and JWA overexpression inhibited proliferation (Fig. 6H, I).

### JWA suppresses tumor growth of trastuzumab-resistant breast cancer by downregulating CDK12 In vivo

**Fig. 7:**
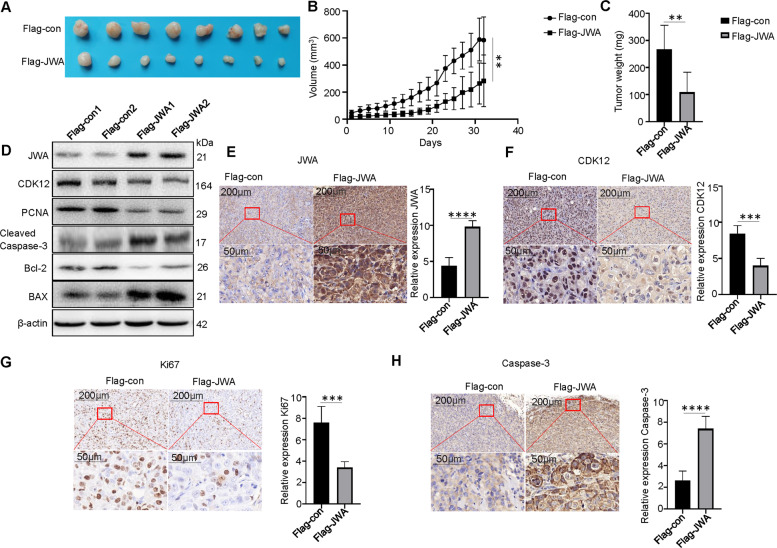
JWA suppresses tumor growth of trastuzumab-resistant breast cancer by downregulating CDK12 In vivo. **A** The xenograft tumors formed by JWA-overexpressed and control vector in JIMT1 cells. **B**, **C** The size and weight of the xenograft tumors formed by JWA-overexpressed and control cells. **D** Protein levels of JWA, CDK12, BAX, Cleaved Caspase-3, Bcl-2, PCNA in tumors formed by JWA-overexpressed and control cells. β-actin served as the internal control. **E**−**H** Images for JWA, CDK12, Ki67, and Caspase-3 staining are shown for the xenografts. The IHC of animal models showed that JWA increased after overexpression of JWA, CDK12 and Ki67 expression decreased, and Caspase-3 expression increased. **P < 0.01, ***P < 0.001, ****P < 0.0001.

Based on our in vitro observations, we sought to validate our data by establishing xenografts in BALB/c nude mouse models. JIMT1 cells with stable JWA overexpression (Flag-JWA group) and control JIMT1 cells (Flag-con group) were subcutaneously transplanted into nude mice. By stripping tumors from nude mice, we presented xenografts from the two groups after 32 days. There was no statistically significant difference in body weight between the JWA overexpression and control groups (Supplementary Fig. 2A). The size and weight of the tumors formed by JIMT1 cells with stable JWA overexpression were significantly decreased compared to those formed by the control group (Fig. 7A−C). The tumor/body weight of the JWA overexpression group was significantly lower than that of the control group (Supplementary Fig. 2B).

We further performed western blotting to detect the protein expression of JWA, CDK12, Bcl-2, BAX, Caspase-3, and PCNA in xenograft tumor tissues. Compared with the tumors formed by control JIMT1 cells, the tumors formed by JIMT1 cells with stable JWA overexpression showed markedly increased JWA, BAX, and Cleaved Caspase-3 protein levels, and decreased CDK12, Bcl-2, and PCNA protein levels (Fig. 7D). These results were similar to the in vitro results. Taken together, these results indicate that JWA suppresses xenograft tumor growth of breast cancer cells by stabilizing JWA in vivo. Moreover, we examined the expression of JWA, CDK12, Ki67, and Caspase-3 in the tumor masses through IHC staining. By conducting an IHC assay using anti-JWA antibody, we found that tumors formed from overexpressed JWA-infected cells exhibited increased expression levels of JWA compared to those in tumors formed from control cells (Fig. 7E). Meanwhile, CDK12 marked tumors overexpressing JWA-infected cells were lighter and less than those in the control group (Fig. 7F), and Ki67 was lower than that in the control group (Fig. 7G), while Caspase-3 was higher than that in the control group (Fig. 7H). Therefore, JWA was able to inhibit breast cancer growth in a mouse model.

## Discussion

HER2+ breast cancer has a poor prognosis [30]. Although the development of anti-HER2 therapies has significantly benefited patients with HER2+ breast cancer, several key issues remain challenging. More than half of HER2+ breast cancer patients do not respond to trastuzumab or develop resistance [8, 31]. However, the mechanisms underlying trastuzumab resistance are still not fully understood. In our study, the data clearly showed that breast cancer tissue had lower levels of JWA than those of normal tissues. Subsequently, the expression of JWA was studied using IHC, which demonstrated that JWA was less expressed in trastuzumab-resistant breast cancer tissues than in trastuzumab-sensitive tissues, suggesting that the downregulation of JWA in trastuzumab-resistant breast cancer might be associated with tumor growth. These findings suggest a potentially important role of JWA in the underlying biological mechanism of trastuzumab-resistant breast cancer. The aim of this study was to identify JWA as a biomarker for trastuzumab-resistant breast cancer cells and to elucidate the underlying mechanisms.

Qiu et al. [32] reported that low expression of JWA in gastric cancer tissues was significantly correlated with shorter OS and advanced clinicopathologic features. Therefore, it is logical to hypothesize that low JWA levels are involved in the pathogenesis of cancer progression. To test this hypothesis, assays were used to investigate the role of JWA in the regulation of breast cancer cell proliferation and apoptosis in vitro and in vivo. The results indicated that overexpression of JWA in JIMT1 and BT474-Tr cells reduced cell proliferation and increased apoptosis of breast cancer cells. In contrast, siRNA-mediated JWA knockdown in JIMT1 and BT474-Tr cells showed the opposite result. These findings indicate that JWA is an important contributor to trastuzumab-resistant breast cancer proliferation and apoptosis.

To investigate JWA-associated downstream molecular events affecting trastuzumab-resistant breast cancer proliferation and apoptosis, RNA-seq was used to compare the mRNA expression profiles of JIMT1 Flag-JWA cells and Flag-con cells, and 16 patients with trastuzumab-resistant breast cancer were tested using NGS. They all had changes in TP53, PIK3CA, CDK12, and PTK2 among genes related to the cell cycle, and CDK12 is known to play a role in regulating cell cycle progression [21]. These results suggest that JWA regulates cell proliferation and apoptosis by regulating CDK12.

Our results showed that CDK12 was more highly expressed in trastuzumab-resistant cell lines than in trastuzumab-sensitive cell lines. High levels of CDK12 have been found in a variety of human tumors and are characterized by uncontrolled cell proliferation [33]. CDK12 is a potential carcinogenic driver and the synergistic effect of CDK12 and HER2 promotes the initiation and progression of HER2+ breast cancer [25]. Capra et al. [34] found that CDK12 expression was upregulated in HER2+ breast cancer and demonstrated a strong correlation between CDK12 levels and high tumor grade. They also suggested that a high CDK12 level could serve as a prognostic marker.

Our study also showed that CDK12 expression was decreased after JWA overexpression in breast cancer cells or tissues. More recently, Chirackal et al. [35] found that inhibition of CDK12 induces G1/S cell cycle progression defects. RNAi treatment with CDK12 significantly increased the number of G0/G1 phase cells, suggesting that CDK12 plays an important role in controlling the transition from the G0/G1 phase to the S phase [36]. Tien et al. [29] found that CDK12 promotes the migration and invasion of HER2+ breast cancer by regulating the alternative last exon splicing of the DDR activator ATM (ataxia telangiectasia-mutated) and DNAJB6 (DnaJ homolog subfamily B member 6). Choi et al. [25] found that CDK12 activates the ErbB-PI3K-Akt or Wnt signaling cascade to drive the initiation of breast tumors and induce trastuzumab resistance. Furthermore, Peng et al. [27] suggested that CDK12 promotes cancer cell stemness, metastasis, and invasion through c-myc / β-catenin pathways.

Based on our results, JWA plays a crucial role in HER2+ trastuzumab-resistant breast cancer by controlling CDK12 expression. In addition, a low expression level of JWA is closely related to poor outcomes, indicating that JWA can be a biomarker of breast cancer prognosis and a promising therapeutic target for cancer treatment.

## Supplementary information

Supplementary figure

## Data Availability

Correspondence and requests for materials should be addressed to Wei Li, Yongmei Yin, or Changqing Wang.
