# Supplementary material for: JWA suppresses proliferation in trastuzumab-resistant breast cancer by downregulating CDK12
Source: Cell Death Discov. 2021 Oct 22;7:306. doi: 10.1038/s41420-021-00693-9 (PMC8536718; doi:10.1038/s41420-021-00693-9)
Supplement: Supplementary file 1 — Supplementary figure [file 41420_2021_693_MOESM1_ESM.pdf]

Supplementary Figure 1

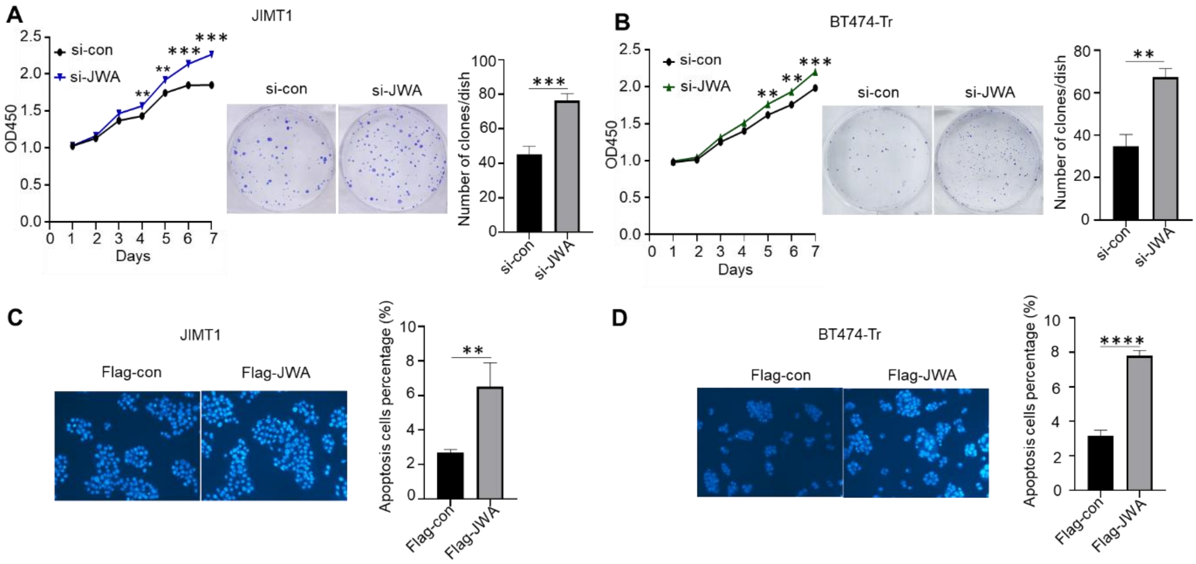

Supplementary Figure 1 JWA inhibits proliferation and promotes apoptosis in trastuzumab resistant breast cancer cells in vitro. (A-B) Knockdown of JWA in JIMT1 and BT474-Tr cells promoted cell proliferation as determined by CCK-8 and colony formation assays. **(C-D)** Apoptosis cell counting analysis of cells transfected with JWA particles, and cell growth was measured using fluorescent photomicrographs to capture the cells with blue fluorescence. ** $P < 0.01$, *** $P < 0.001$, ****$P < 0.0001$.

Supplementary Figure 2

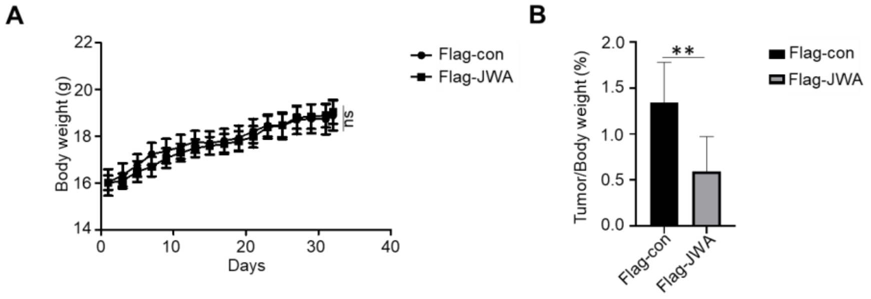

Supplementary Figure 2 JWA suppress tumor growth of trastuzumab resistance breast cancer by downregulating CDK12 In vivo. **(A)** There was no statistically significant difference in body weight between JWA overexpression and control mice. **(B)** The tumor/body weight of JWA overexpression group was significantly lower than that of control group. ** $P < 0.01$, ns: statistical difference.
